# TUM4Health, a holistic student health promotion program. Screening of cardiovascular risk factors in university students

**DOI:** 10.3389/fcvm.2024.1428457

**Published:** 2024-11-27

**Authors:** Klaus Christian Haggenmüller, Barbara Reiner, Renate Maria Oberhoffer, Nils Olson, Jochen Weil, Thorsten Schulz

**Affiliations:** ^1^ Chair of Preventive Pediatrics, Technical University of Munich, Munich, Germany; ^2^ German Heart Centre Munich, Hospital of the Technical University of Munich, Munich, Germany

**Keywords:** cardiovascular risk factors, ACCF/AHA practice guideline, sports & exercise medicine, CPET cardiopulmonary exercise testing, echocardiography, electrocardiography (ECG), biomarkers

## Abstract

Cardio Vascular risk prevention in Germany has a gap between the ages of 20 and 30 years. We established a program for risk group identification in students and analyzed the screenings according to the ACCF/AHA Stages and NYHA functional classification. In a cross-sectional design, 596 participants completed a sports medical and motor performance check-up. We found 6% of the female subgroup in an underweight status. Low blood pressure in 5% of the male and 10% of the female group. Elevated blood pressure in 27% of the male and 12% of the female subgroup. According to ACCF/AHA classification, a percentage of 25.7% were clustered in Stage A, defined as having a high risk for heart failure (HF). Stage B consisted of participants with structural heart disease but without symptoms of HF, the prevalence of this group was 3.2%. In Stage C we assigned 0.7%, respectively four participants, with structural heart disease and prior symptoms (NYHA Stage C1 and C2). Screenings sensitize CV risk factors and detect HF risks at an early age; for freshmen at universities it seems to be the perfect time and place for secondary prevention. The gap in risk prevention can be closed, at least for students, who are an economically important target group. Moreover, we found a higher prevalence of Stage C in the sports-related study population compared to other studies. The risk for HF could be elevated in sports-related faculties. The high prevalence of underweight participants, especially in the female subgroup could also be a specific problem in sports-related groups. Even if not related to CV risks, the awareness of being underweight in combination with offers for a lifestyle change can prevent risks in the future. The same applies to low blood pressure in the female subgroup.

## 1 Introduction: background and aims

### 1.1 Background

Untreated cardiovascular (CV) risk factors can lead to serious health problems and are responsible for one-third of overall mortality in Germany (1). In Germany, the first diagnosed ischemic heart diseases occur more frequently in men aged 40 and above, whereas in women, they typically occur from the age of 50 onwards (2), thus appearing in the middle adulthood Stage, spanning from 35 to 65 years old. Secondary prevention programs are needed to detect CV risk factors and sensitize young adults [19–39 years, (3)] to a preventive lifestyle. Overall, the largest proportion of the university students in Germany belong to this early adulthood group respectively the “young adult” group.

In Germany, public health insurances provide secondary prevention for children, which ends at the age of 14 (Preventive medical check-up J1). On average, only 47.7% of students participate in this last free prevention examination (4). At the age of 17, another preventive offer (J2) is available, but with costs, and only 17% of students make use of it (4). That is why about half of the 473.00 first-year university students in Germany with an average age of 21.9 years (5) at German universities are therefore among the “young adults”, who had their last preventive examination eight years ago before starting their studies. Some even had their last obligatory screening at the age of six, i.e., sixteen years before starting their studies.

Nonetheless, a striking number of “young adults” in Germany were found to have cardiovascular precursors Stage A of heart failure according to AHA/ACCF guidelines and increase with advancing age. In the Morbach et al. study population, an anatomical change in the heart (Stage B risk factor) visible through ultrasound was conspicuously found in “young women” even regardless of Stage A risk factors (6). Moreover, the results showed the need for screenings at an early age to cope with risk factors and avoid circumstances that could result in cardiovascular events.

There is a lack of emphasis on preventive healthcare for individuals aged 20–30. Offering CV screenings to this age group may close this gap and improve the prevention of CV risks. Detecting heart disease is crucial for maintaining good health in later years, as most cases of sudden cardiac death in middle-aged men are additionally linked to recreational sports (7). In a subsequent study, Bohm et al. found that coronary artery disease is the leading cause of sports-related sudden cardiac arrest in young adults, especially in recreational male sports (8). Controversially, when practicing the recommended preventive measures of recreational sports. However, physical fitness, best initiated through regular physical activity, is a major factor in supporting cardiovascular health in combination with a healthy lifestyle (9).

Although students represent an important socio-economic group in both the short and long term, they are often neglected in terms of secondary prevention programs. Therefore, to identify potential health issues among university students, draw attention to cardiovascular risk factors, and promote a healthy lifestyle, we initiated a health-promoting program at the TUM called TUM4Health. It includes a health-related screening, similar to the pre-participation screening of athletes in professional sports, to identify multiple risk factors, with a focus on CV risk factors. According to Aberg et al., the cardiovascular fitness of 18-year-olds is also closely linked to their cognitive performance (10). Likewise, aerobic fitness and muscular endurance seem to positively affect academic achievement in middle school students or university students (11, 12). This means that cardiovascular fitness can be used to predict their future educational success and socio-economic status. Therefore, making lifestyle changes at an early stage can help reduce CV risk factors and possibly decrease the likelihood of cardiovascular events. Additionally, it can also contribute to better mental and cognitive health. That is why universities may provide an ideal setting for CV prevention programs.

The American College of Cardiology Foundation (ACCF) and the American Heart Association (AHA) have jointly produced guidelines for the categorization of cardiovascular disease, respectively heart failure (HF) since 1980 (13). Additionally, the New York Heart Association (NYHA) defined functional classifications for each of the stages (13). These stages and functional classifications are commonly used to have comparable results in the risk group definition.

### 1.2 Aims

This study aims to provide an epidemiological perspective on the health status of young adults at a university. The main research question is determining the prevalence of undetected CV risk factors in this population according to the ACCF/AHA stages and NYHA functional classification. Specifically, we want to identify health-related physical fitness factors that impact the health of this study population and the percentage of students affected by health-related risk factors. To achieve this, we conducted cross-sectional screenings to collect medical health parameters such as detailed medical history, body composition, echocardiography, resting ECG, exercise ECG, exercise blood pressure, lung function, and questionnaires. Additionally, we also collected sports-related parameters such as strength, speed, and reaction.

## 2 Methods

The cross-sectional screening was offered to students in the first two semesters. All first- and second-semester students were invited to participate in this study free of charge and on a voluntary basis. Participation or non-participation had no impact whatsoever on their further studies. Since there was more interest than available appointments, a first-come, first-served basis was applied.

Inclusion criteria were enrolment at TUM in the bachelor programs Sport Science or Health Science. Exclusion criteria were known pre-existing conditions that contradict an examination especially on the CPET (e.g., acute knee or ankle injuries, pneumothorax, chronic obstructive pulmonary disease), acute illness on the day of the test that prevents participation from a medical point of view, or age under 18 years.

Between October 2017 and February 2024, 645 students participated in the screenings. A group of 31 Participants attended the medical part but missed the motor performance tests. Another 17 participants did not answer the questionnaires and one participant withdrew his consent. Finally, 596 participants were included in this study. The examination team consisted of a sports scientist, a cardiologist, and research assistants.

The test battery consisted of non-invasive diagnostic methods of cardiovascular, pulmonary, and motor performance. Additionally, the participants had to fill in different questionnaires. The local ethical board of the Technical University of Munich (Ethical Board Number 379/19 S-SR) has approved the program. All participants signed a consent form for the examination and the anonymized usage of the data for scientific research.

Students who displayed acute health risks during the examination were advised to consult a specialist. For students with potential risk factors, we offered participation in TUM4Health programs to promote a healthy lifestyle.

### 2.1 Sports medical check-up

The focus of the medical screening was on cardiovascular prevention. All (sports) medical examinations were non-invasive and conducted under medical supervision. The participants were instructed by E-mail to consume regular food and drinks before the medical check-up. No specific nutritional protocol was followed.

#### 2.1.1 Anthropometry and medical history

The anthropometric data including body composition was measured by bioimpedance body composition analysis using the system “InBody 770” (InBody Europe B.V., Eschborn Germany). Bioimpedance is highly correlated with the gold standard DEXA scans (14) but has no impact on the test subjects. The subjects did not follow any dietary instructions in advance. The focused parameters were body mass, skeletal muscle mass, body fat, and body size.

A physician collected the medical history with standardized questions about sports history, personal medical history, previous illnesses, and family history.

#### 2.1.2 Blood pressure (BP)

The blood pressure measurement, the pulse wave velocity, and the central blood pressure were performed on the left arm brachial artery in rest, lying position using a Mobil-O-Graph (I.E.M., Stolberg Germany). The Mobil-O-Graph system works automatically, is validated, and is frequently used (15), the second measurement was used for the analysis. At rest, we measured the peripheral and central blood pressure as well as the pulse wave velocity after at least 10 min of lying at rest. We used the thresholds for risk values from the American Heart Association (16) and for pulse wave velocity the analysis of Inuzuka et al. (17).

Throughout the CPET on a bicycle ergometer, we measured blood pressure on the left arm by Suntech Tango M2 (SunTech Medical Inc., Morrisville USA) before the start, end of the 3 min warm-up, after 3 min, and after 6 min on the ramp, at maximum and 1 and 3 min after abort. The Suntech system works for measurements under exercise with high precision (18).

#### 2.1.3 Electrocardiography (ECG)

We performed a 12 leads electrocardiography (19) for one minute after lying down for at least 5 min in rest and the whole time of the CPET by custo ergo diagnostic 300 (custo med GmbH, Ottobrunn, Germany). All ECG were screened for signs of ventricular hypertrophy as well for the detection of anomalies of repolarisation such as the long QT syndrome or for potential life threatening arrhythmias such as the Brugada's syndrome.

#### 2.1.4 Echocardiography (UCG)

A cardiologist performed the echocardiography with a GE Vivid E9 XDclear (GE Healthcare GmbH, Munich Germany). The biomarker LVPWd measures the myocardial thickness of the left ventricle. We used the left ventricle posterior wall thickness in diastole (LVPWd), with a normal range of 6–12 mm (20). For the echocardiographic examination the following standard views were used: parasternal long and short axis, apical 4- and 2 chamber view, suprasternal short and long axis, subcostal view as well the deliniation of abdominal situs with the aorta in long- and short axis view. To measure left ventricular wall thickness, we used M- mode echocardiography in the parasternal long axis view.

#### 2.1.5 Cardio-pulmonary-exercise-test (CPET) and lung function (LF)

At rest, the participants performed a lung function test. The lung function (LF) was measured as described by Quanjer et al. as a Tiffenea*u*-Test (21) using the custo spiro mobile device (custo med GmbH, Ottobrunn, Germany). The main parameters were the forced vital capacity (FVC), inspiratory vital capacity (IVC), and relative forced expiratory vital capacity (FEV1%) (22, 23). The standard values for the analysis were used according to the studies performed by Bauer (24) or Quanjer et al. (21). For the analysis the best out of three tries was used.

We used the ergometer Excalibur (Lode B.V., Netherlands), connected to the measuring device Metalyzer 3b (Cortex Biophysik GmbH, Germany). All participants performed the same test protocol on the ergometer. The ramp protocol started with a 3-minute recording of the rest parameters, then 3 min warm up with 50 Watt. The ramp continued at 50 watts and increased the resistance continuously by 20 Watts per minute. The test results were analyzed in the “MetaSoft Studio” software using the “Wassermann 9 Panel Plot” (25). The main outcome of this study was the VO2peak. This is defined as the maximum point of the O_2_ consumption (25). We compared the measurements with standard values of maximum oxygen uptake (26, 27). Other markers were the maximum power outcome (Pmax) and the maximum heart rate (HRmax).

### 2.2 Motor performance check-up

Sports motor performance tests are widely used procedures to evaluate motor performances in sports and healthcare settings, as well as to identify any possible deficits such as movement restrictions or postural defects. These tests involve maximum muscular exertion. The detailed test descriptions are documented in the additional material.

### 2.3 Risk group stages (ACCF/AHA) and functional classification (NYHA)

According to the classification of the ACCF/AHA and the functional classification of NYHA (13), this study classified risks of heart failure in Stages A to D (see Table 1).

**Table 1:** Risk group stages (ACCF/AHA) and functional classification (NYHA) [illustration according to (13)].

Risk group stages (ACCF/AHA) and functional classification (NYHA)			
ACCF/AHA risk group		NYHA classification	
Stage A (one or more)	• Elevated blood pressure • Obesity • Smoking • Alcohol or substance abuse		
Stage B	Structural heart disease but without signs or symptoms of Heart failure (HF)	I	Asymptomatic and no limitations of physical activity
Stage C	Structural heart disease and prior or current symptoms of HF	II	Ordinary physical activity does not cause symptoms of HF
		III	Ordinary physical activity results in symptoms of HF
Stage D	Refractory HF requiring specialized interventions	IV	Symptoms at rest, inability to carry out physical activity without malaise

ACCF, American College of Cardiology; AHA, American Heart Association; NYHY, New York Heart Association.

Participants in Stage A, are defined as having a high risk for HF but have no structural heart disease or symptoms of HF. Stage A has no functional classification by the NYHA., this study basically assigned the following risk factors in this stage according to the meta-analysis of Barbaresko et al. (9). In difference, we collected no blood samples, and in the obesity group, we excluded participants who were overweight but with high muscle mass. The definition of critical alcohol consumption was carried out according to the German Society for Nutrition (28) which is in line with the systematic review in the Lancet about alcohol consumption (29).

1. High Blood Pressure: Elevated blood pressure that can strain the heart over time and can lead to heart failure. Included were participants with hypertension 1 and 2 according to risk classification from the American Heart Association (16).
2. Obesity: Excess weight puts strain on the heart and increases the risk of HF. In the first step participants with a BMI over 25 were included. In the second step, all males with a body fat below 12% and females with body fat below 20% were removed from this class. This subgroup had high weight because of the elevated muscle mass, and this is no CV risk factor.
3. Smoking: Regularly Tobacco use damages the heart and blood vessels and so increases the risk of heart failure. All smokers were included in this stage.
4. Alcohol and Substance Abuse: Critical alcohol consumption and use of drugs can weaken the heart muscle and contribute to heart failure. Participants with regular consumption were included. Rarely use of alcohol was not included.

Participants with structural heart disease but without signs or symptoms of HF were assigned to Stage B and the NYHA class I. The participants in this group had no limitation of physical activity and were allowed to perform the CPET and motor performance tests. Ordinary physical activity does not cause symptoms of HF in this group. Structural heart disease refers to abnormalities or defects in the structures of the heart, including the valves, chambers, walls, and blood vessels. These abnormalities can be present at birth (congenital) or develop over time due to various factors such as aging, infection, inflammation, or other medical conditions (13). For the classification, the left ventricle posterior wall thickness in diastole (LVPWd) was used in combination with the ECG and UCG analysis of the cardiologist.

In Stage C the participants with structural heart disease and prior or current symptoms of HF were assigned. Stage C differs in NYHA functional classes II and III.

C II: This class has no limitation on physical activity, so the participants were allowed to participate in CPET and motor performance tests. Ordinary physical activity does not cause symptoms of HF. The cardiologist assigned participants to this class using ECG, UCG, and preliminary findings.
C III: Here participants have slight limitations of physical activity. They feel comfortable at rest, but ordinary physical activity results in symptoms of HF. In this group, the CPET and motor performance tests were performed. In addition to C I the participants had abnormalities in these tests.

**Figure 1:**
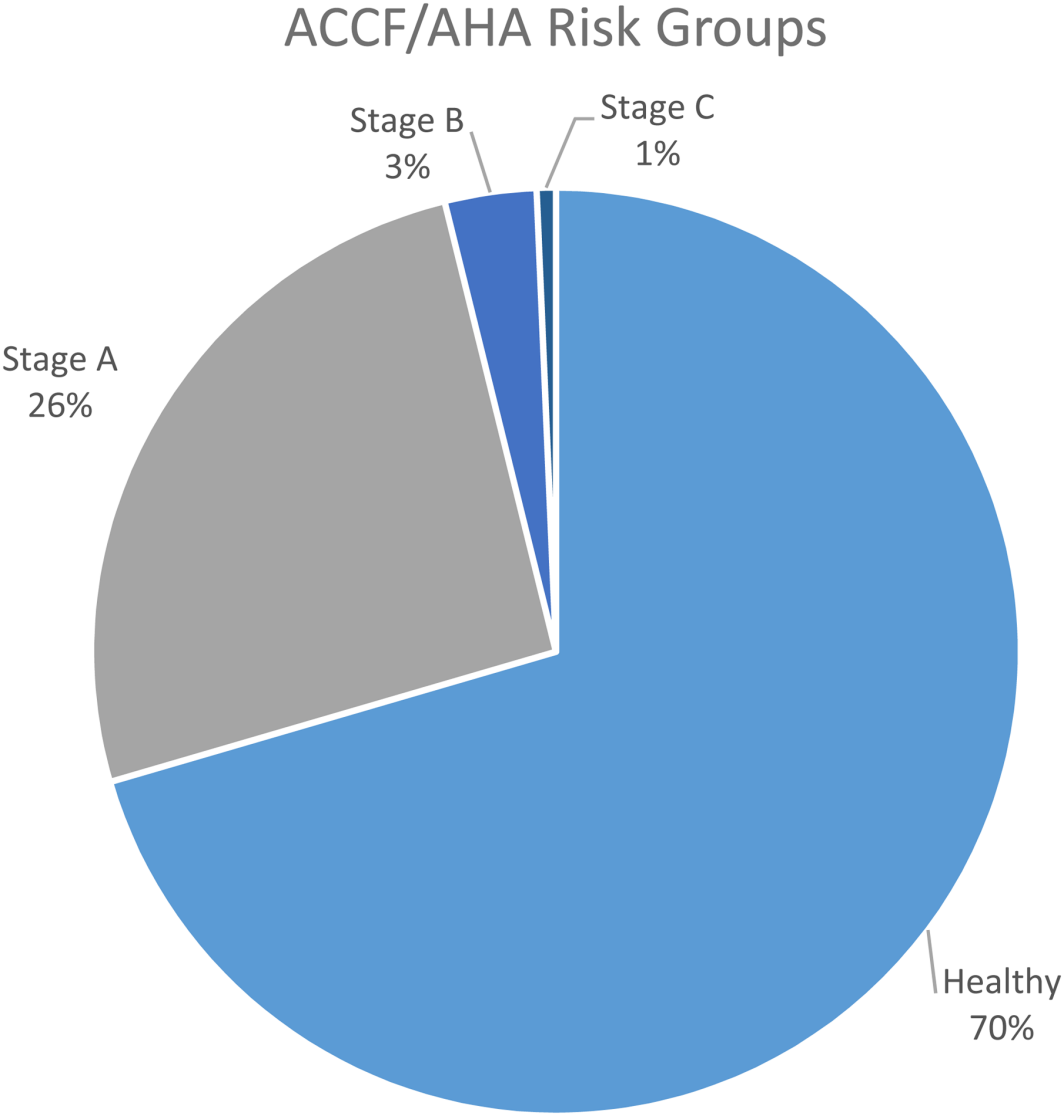
ACCF/AHA risk groups.

In Stage C functional class III, and Stage D (Figure 1), the cardiologist would have categorized according to the ACCF/AHA and NYHA guidelines (9, 13). These stages had no relevance to his study because none of the participants had to be assigned to this category.

### 2.4 Data management and statistics

To carry out the statistical analysis in our study, we utilized the Statistical Package for the Social Sciences (SPSS) software, version [Version 29.0.2.0 (20)], provided by IBM (Armonk, USA). The software enabled us to perform a range of statistical procedures to interpret the data effectively. For the descriptive analysis, we used averages and standard deviations to summarize the central tendencies and variability of our dataset, providing a clear overview of the data distribution.

For inferential analysis, we applied *T*-tests when comparing the means between two independent groups. This allowed us to determine whether there were statistically significant differences between the groups being compared. In cases where more than two groups were involved, we employed Analysis of Variance (ANOVA) tests. ANOVA was crucial for identifying any significant variations across multiple groups, providing insights into the overall trends without inflating the risk of type I error.

Additionally, Pearson's Correlation Coefficient was used to assess the strength and direction of linear relationships between different biomarkers, particularly in the heat map analysis. This helped us identify potential associations and patterns between the variables, further enhancing our understanding of the data and contributing to the overall findings of the study.

## 3 Results

The students were aged 21, ±3 years, with 37% being male. The male participants had an average height of 181, ±7.5 cm and an average weight of 76, ±10 kg. The female participants had an average height of 167, ±6 cm and an average weight of 60, ±8 kg. Overall, 7% of the students stated that they were smokers, with 6% being female and 8% being male.

### 3.1 Descriptive analysis of screening values

Table 2 displays the average and the standard deviation of the screening values. In the last two columns, you can find the number of valid values for male and female participants.

**Table 2:** Screening values medical check-up.

TUM4Health	Male		Female		Male	Female
Medical screening	Average	Standard deviation	Average	Standard deviation	*n*	*n*
BMI [kg/m^2^]	23	±2	21	±2	220	376
Body fat [%]	12	±4	21	±6	214	358
Skeletal muscle mass [kg]	38	±6	26	±4	214	358
IVC% [% norm value]	97	±11	100	±12	168	312
FVC% [% norm value]	99	±12	96	±11	189	339
FEV% [% norm value]	97	±11	95	±10	189	339
VO2peak [ml/min/kg]	50	±7	41	±6	203	241
Pmax [kg]	305	±47	214	±36	192	325
Central blood pressure systole [mmHg]	117	±12	106	±9	199	335
Central blood pressure diastole [mmHg]	73	±9	72	±8	200	335
Pulse-wave [m/s]	5.3	±0.5	4.9	±0.3	193	334

BMI, body mass index; IVC, inspiratory vital capacity; FVC, forced vital capacity; FEV, forced expiratory volume; Pmax, power maximum.

Table 3 presents the results of the motor performance test in an identical structure.

**Table 3:** Screening values motor performance check-up.

TUM4Health	Male		Female		Male	Female
Motor skills	Average	Standard deviation	Average	Standard deviation	*n*	*n*
Counter movement jump [cm]	39	±6	26	±4	151	266
Drop jump [cm]	27	±9	21	±6	152	266
Handgrip right [kg]	49	±8	31	±5	150	260
Handgrip left [kg]	48	±9	31	±5	149	259
Tapping [contact]	65	±11	57	±9	154	268
Standing long jump [cm]	222	±41	172	±28	127	242
Plank [s]	204	±79	166	±83	126	240

### 3.2 Prevalence of undetected cardiovascular risk factors

Tables 4–6 display the thresholds for potential risk values of the main screening factors, along with the corresponding number of participants.

**Table 4:** Prevalence of BMI, fat, lung function, and VO2peak.

TUM4Health	Male			Female			Male	Female
Medical Screening	Threshold	*N*	%	Threshold	*N*	%	*n*	*n*
BMI [kg/m^2^] underweight	<18,5	2	1%	<18,5	23	6%	220	376
BMI [kg/m^2^] overweight	25–30	43	20%	25–30	17	2%	220	376
BMI [kg/m^2^] obesity	>30	4	2%	>30	0	0%	220	376
Body-Fat% [%] low	<12%	129	60%	<20%	90	25%	214	358
Body-Fat% [%] high	>20%	8	4%	>30%	12	3%	214	358
IVC% [% norm value]	<80%	6	4%	<80%	9	3%	168	312
FVC% [% norm value]	<80%	7	4%	<80%	20	6%	189	339
FEV% [% norm value]	<80%	6	3%	<80%	19	6%	189	339
VO2peak% [% norm value]	<80%	4	2%	<80%	9	3%	203	341
LVPWd [mm]	>12	7	3%	> 12	16	4%	205	360

BMI, body mass index; IVC, inspiratory vital capacity; FVC, forced vital capacity; FEV, forced expiratory volume; LVPWd, left ventricular posterior wall diameter.

**Table 5:** Prevalence hypertension.

TUM4Health	Male			Female			Male	Female
Medical Screening	Threshold	*N*	%	Threshold	*N*	%	*n*	*n*
Blood Pressure elevated: systole 120–129 and diastole less than 80 [mmHg]	120–129 <80	57	27%	120–129 <80	45	12%	214	364
Blood Pressure hypertension stage 1: Systole 130–139 or diastole 80–90 [mmHg]	130–139 80–90	41	19%	130–139 80–90	14	4%	214	364
Blood Pressure hypertension stage 2: Systole >139 or diastole >90 [mmHg]	>139 >90	23	11%	>139 >90	5	1.4%	214	364
Central Blood Pressure elevated: systole 120–129 and diastole less than 80 [mmHg]	120–129 <80	44	22%	120–129 <80	17	5%	199	335
Central blood pressure hypertension stage 1: Systole 130–139 or diastole 80–90 [mmHg]	130–139 80–90	20	10%	130–139 80–90	5	1.5%	199	335
Central blood pressure hypertension stage 2: Systole >139 or diastole >90 [mmHg]	>139 >90	10	5%	>139 >90	5	1.5%	199	335

**Table 6:** Prevalence hypotonic, pulse-wave, ECG, and cardiac ultrasound.

TUM4Health	Male			Female			Male	Female
Medical screening	Threshold	*N*	%	Threshold	*N*	%	*n*	*n*
Blood pres ure low systole <100 or diastole <60 [mmHg]	<100 <60	11	5%	<100 <60	36	10%	214	364
Central blood pressure low systole <100 or diastole <60 systole [mmHg]	<100 <60	19	10%	<100 <60	76	23%	199	335
Pulse-wave [m/s]	>8.2	0	0%	>8.2	1	0.3%	193	334
ECG rest noticeable	Physician	34	15%	Physician	47	13%	220	376
ECG exercise noticeable	Physician	44	20%	Physician	60	16%	220	376
Cardiac Ultrasound noticeable	Physician	39	18%	Physician	51	14%	220	376

ECG, electrocardiogram.

In the male subgroup, 44 participants had a BMI above the normal threshold, while 129 participants had a low percentage of body fat. Two male participants were underweight. In the female subgroup, 23 participants were underweight and 90 had a low percentage of body fat. None of the female participants were in the BMI obesity category, but 12 had a high body fat value. A relatively high number of participants of both sexes were below 80% in at least one of the comparative values in lung function and VO2 peak compared to average values of the NHANES III survey in the US (30).

According to the American Heart Association, we defined three categories for abnormal blood pressure by combining systole and diastole measurements (16). Table 4 illustrates the number of participants in each category, including elevated, hypertension Stage 1, and Stage 2. In the male subgroup, hypertension is more common. Additionally, the peripheral measurement showed higher values in all categories than the calculated central blood pressure.

It was found that hypotension was more common among females. In addition, one female participant had a pulse-wave velocity above the threshold. A cardiologist evaluated the electrocardiogram and found similar percentages of noticeable values for both male and female participants. Lastly, in the echocardiography, slightly more males had noticeable findings. Overall parameters 42.2% of the study population had elevated values in at least one of the screening values.

### 3.3 Risk group stages (ACCF/AHA) and functional classification (NYHA)

According to the classification of the ACCF/AHA and the functional classification of NYHA (13), this study identified risks of heart failure in Stages A to C. 176 participants were assigned in risk groups, the prevalence over all stages was 29.5% (*n* = 596).

The prevalence in Stage A was 25.7% (153 participants). The risk factors in Stage A were:

1. High Central Blood Pressure (Hypertension Stage 1 and Stage 2): 40 participants, 6.7%
2. Obesity (BMI over 25): 64 participants, 10.7%
3. Smoking regularly: 41 participants, 6.9%
4. Alcohol and Substance Abuse: 32 participants, 5.4%

The prevalence in Stage B was 19 participants (3.2%). In Stage C, functional classification II one person was assigned and in III three persons (Figure 1).

**Figure 2:**
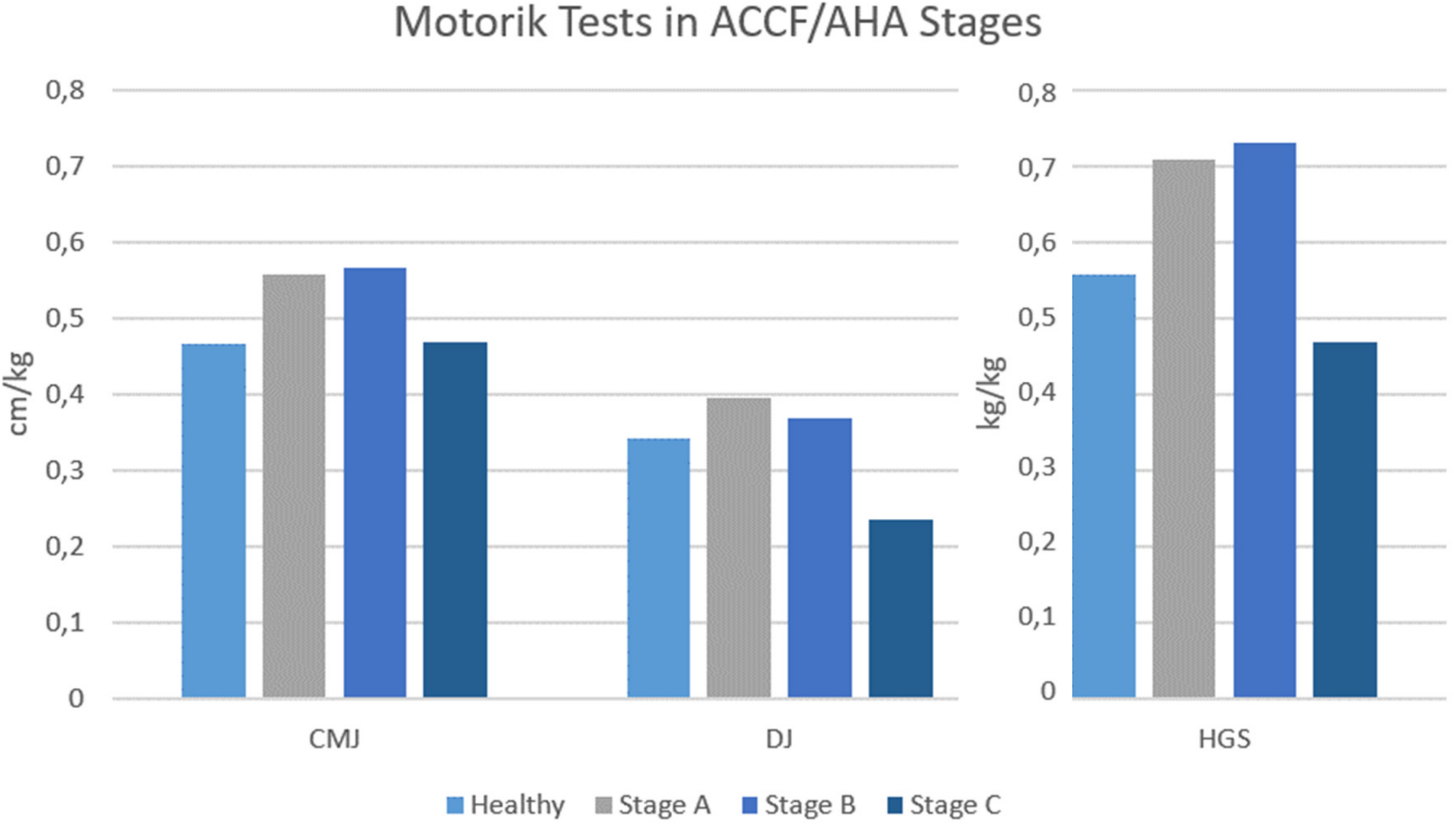
ACCF/AHA stages compared to healthy in countermovement-, drop-jump, handgrip-strength.

The analysis of the motor performance test results between the risk groups was predominantly not significant. The counter movement jump and the drop jump, shown in Figure 2 adjusted to kg bodyweight, had differences in the mean values between the risk groups but were not statistically significant. One significant difference was found in the mean values of HGS per kg bodyweight between the ACCF/AHA groups (F(4,408) = 10,487, *p* < .001, *η*p^2^ = .435, *n* = 50). (Figure 2).

### 3.4 Associations between the biomarkers

The correlations between the screening items are presented in Supplementary Table S1 as Pearson correlation coefficients. High positive correlations are shown in dark blue, while high negative correlations are shown in bright red. The brighter the correlation, the lower it is. We summarized the items in 6 main factors: anthropometry, blood pressure, decisions by the physician, lung function values, CPET, and motor performance parameters. The anthropometric values were correlated with each other with a significance level of *p* < 0.001. Also, the Blood pressure parameters including pulse wave velocity were correlated with *r* = 0.363 up to *r* = 0.924, and all of them were significant with *p* < 0.001. The values collected or interpreted by the physician were almost independent of each other. High correlations were between the lung function parameter (Pearson correlation; IVC% to FVC%, *r* = 0.899, *p* < 0.001, 480; IVC% to FEV%, *r* = 0.710, *p* < 0.001, 480; FEV% to FVC%, *r* = 0.761, *p* < 0.001, 528). The CPET values were indifferent. VO2peak in L/min and ml/min/kg were high and significantly correlated (*r* = 0.788, *p* < 0.001), Pmax, maximum heart rate, and blood pressure in exercise were independent. Another block was the strength parameter CMJ, DJ, HGS, and SLJ, with *r* = 304 up to *r* = 908 and all significant with *p* < 0.001. The plank test and the tapping test were standing alone.

A general marker was fat tissue in percentage, which was significantly negatively correlated with values of all six main factors (*p* < 0.001). Other markers that were positively correlated over the six main factors were VO2peak in L/min, Pmax, CMJ and HGS left and right. This marker had the fewest correlations with all other parameters of this study.

As already known sex and age are critical factors that can influence the relationships of parameters. Most of the values were already adapted to sex and age differences by setting them in relation to norm values. For other relevant values, partial correlation analysis were used for a more accurate understanding of their relation. (Table 7). The identified assossiations persisted even when taking age and gender into account.

**Table 7:** Partial correlations.

Partial correlation sex and age	Fat tissue [%]	Central systole in Rest [mmHg]	Central diastole rest [mmHg]	LVPWd [mm]	VO2peak [L/min]	CMJ [cm]	DJ [cm]	Handgrip strength [kg]	Tapping	Standing longjump [cm]
Fat tissue [%]	1.000	0.031	0.085	0.001	−0.145	−0,315^b^	−0,187^b^	−0.095	0.068	−0,272^b^
Central systole in Rest [mmHg]	0.031	1.000	0,565^b^	0.046	0.156	0,181^a^	0.174	0.063	0.005	0.088
Central diastole rest [mmHg]	0.085	0,565^b^	1.000	−0.100	−0.153	0.107	0.122	−0.057	0.053	0.040
LVPWd [mm]	0.001	0.046	−0.100	1.000	0.155	0.010	0.027	0.089	0.065	0.075
VO2peak [L/min]	−0.145	0.156	−0.153	0.155	1.000	0,243^b^	0.077	0,392^b^	−0.090	0.151
CMJ [cm]	−0,315^b^	0,181^a^	0.107	0.010	0,243^b^	1.000	0,466^b^	0,259^b^	0,202^b^	0,512^b^
DJ [cm]	−0,187^a^	0.174	0.122	0.027	0.077	0,466^b^	1.000	−0.078	0.085	0,276^b^
Handgrip strength [kg]	−0.095	0.063	−0.057	0.089	0,392^b^	0,259^b^	−0.078	1.000	0.064	0,185^a^
Tapping	0.068	0.005	0.053	0.065	−0.090	0,202^b^	0.085	0.064	1.000	0.098
Standing longjump [cm]	−0.273	0.088	0.040	0.075	0.151	0,512^b^	0,276^b^	0,185^a^	0.098	1.000

LVPWd, left ventricular posterior wall diameter; CMJ, counter movement jump; DJ, dropjump.

^a^ Significant <.001.

^b^ Significant <.0001.

## 4 Discussion

We suggested that young adults studying sports and health science might have a lower risk of health issues due to their more active lifestyle compared to the general population. The analysis of the motor performance parameters confirmed this assumption for the study population, which was in line with the literature (31). Nevertheless, the non-categorized descriptive analysis of the screening parameters showed a prevalence of elevated risk factors in 42.2% of the participants. This value included the slightly elevated screening parameters. The analysis of risk group stages of ACCF/AHA and functional classifications of NYAH structured the results to identify cardiovascular risk factors.

### 4.1 The health status of the study population—an epidemiological perspective of screening parameter

The average BMI was within the normal range, and their percentage of body fat was close to the lower threshold of healthy values (8, 32). In terms of body composition, the amount of skeletal muscle mass was above normal values, while the proportion of fat tissues was below normal values, which is typical for a sporty population. The average values indicated a healthy and sports-oriented group. However, the female subgroup showed a significant issue with an underweight of 6%. Many females had low body fat, but this could be offset by higher muscle mass. The BMI provided valuable information about the number of underweight participants in the female subgroup, as it accounted for the fat-muscle shift and body size. These findings basically align with Roh et al., who also found a high prevalence of underweight in females and associated health risks with it (33). However, the percentage of underweight individuals in our population was twice as high. However, a recent study also found an elevated prevalence in regards to underweight among female students within the subject fields of sports and health sciences (34). Underweight is a concern for a sports-oriented population and can impact future health. On the other hand, overweight, as indicated by the BMI, was mainly present in the male population. The high muscle mass in the population could lead to misleading conclusions. This limitation of the BMI, especially for strength athletes, is well-documented (35). The obesity category applied to our population but had a low prevalence of 2%. The body mass index worked well for female participants to detect underweight status, which was common in this subgroup. For the male subgroup, the BMI worked in the obese and underweight categories. The overweight category was mainly affected by the elevated muscle mass of the men and had no prognostic value for future health. The lower reference values for fat tissue were not valid for this population; the threshold was too low for physically active individuals. However, the reference value for high fat tissue worked well for both sexes.

The average blood pressure values were within the normal range, as were their pulse-wave values and calculated central values. The highest prevalence of all markers was found in the male subgroup for blood pressure, with 57%, while the female subgroup had a prevalence of 18%. The classification was done according to the American Heart Association (16). The first group, “elevated blood pressure”, had the highest prevalence in both males and females, with 45 women and 57 men. This raises questions about whether this large group has an elevated risk for future cardiovascular events. Although the brachial artery measurement is accepted as an important predictor, there is evidence that central blood pressure measurements are more closely related to future cardiovascular events than brachial artery measurements (36). The central measurement had a prevalence of 31% in males and 4% in females, which seemed to be more suitable for individual risk assessments. In the group with low blood pressure, the prevalence between brachial artery and central measurements was the other way around, with the central measurement. The prevalence of low blood pressure was 23% in females and 10% in males. Low blood pressure is not necessarily an indicator of cardiac arrhythmias, nevertheless, long-term health problems can occur. The pulse wave velocity showed only one woman with an elevated value. Hypertension in stages 1 and 2 was more prevalent in the male subgroup, while hypotension was more prevalent in the female subgroup.

The individual's lung function values, including inspiratory, expiratory, and one-minute expiratory vital capacity, were all within the normal range. Their VO2peak values were in the range of semi-professional athletes. The lung function and CPET results also confirmed the active lifestyle. The average markers for lung function were in the high-normal range, and the CPET test on the bicycle ergometer provided values similar to semi-professional athletes, especially in VO2peak and Pmax. These results confirmed the aerobic capacity of the participants was higher than in peer groups. The lung function test showed low values of 4% in male and 6% in female participants. This is higher than the estimated prevalence of low lung function for the age group 17–24 years, which was 2.9% in males and 3% in females according to a study in the US (NHANES III) (30). Despite a lower proportion of smokers (7%) in our study compared to the normal population in the US (29%), the prevalence of low lung function was surprisingly higher. Further medical examinations were recommended for participants with low lung function, as early detection of lung problems can reveal long-term health issues. We conducted a test to measure aerobic capacity using a CPET, focusing on the main value VO2peak. 4 men and 9 women scored below 80% of the standard values. This, combined with the ECG under exercise and the cardiac ultrasound, led the cardiologist to recommend further examinations. CPET diagnostics are particularly useful for detecting early heart failure with preserved ejection fraction and exercise-induced pulmonary hypertension (37). A study among Polish students aimed to compare different levels of aerobic capacity to cardiovascular risks. The results identified the high VO2max group as a lower risk for CV risks in the future (38). VO2peak is an important predictor of future health.

The examination team included a cardiologist who evaluated the ECG at rest and during the CPET. The most common findings were arrhythmia, ventricular extrasystole, T-Wave abnormalities, and changes in the QRS complex. The prevalence of these findings was higher in the male subgroup compared to the female subgroup, affecting between 13% and 20% of the participants. Overall, 90 deviations were documented, but only 20 were subsequently classified as conspicuous and in need of control. In the analysis of the Cardiac Ultrasound, we focused on the value of myocardial tissue thickness of the left ventricle, as an abnormal higher left ventricle mass could be an early sign of hypertensive heart disease (39). 4% of the participants showed elevated values.

The muscular parameters were close to the requirements for professional athletes. The average jump height in CMJ of the male group was one centimeter above the prerequisite jump height, set by the German football association for young athletes. The male participants performed higher across all parameters compared to their female counterparts. In the male category, the CMJ values exceeded 40 cm on average. In the guideline for professional football players in Germany, 40 cm is the requirement for male athletes. (40). The average handgrip strength for men in this age group is 47.6 kg, while for women it is 30.5 kg, according to Schipf et al. (41). Our participants surpassed this average value of normal population in handgrip strength. It is also noteworthy that in the standing long jump, our participants would achieve an average bronze category rating according to the German sports Badge table (42). The study results show, that even a physically active population is not immune to cardiovascular events and health issues.

### 4.2 CV risk groups (ACCF/AHA)

As expected, the healthy group was the largest in our population. The study by Morbach et al. also classified the participants in the ACCT/AHA systematic and found in their youngest age group (30–39 years) nearly the same percentage of healthy participants. With increasing age, this changed significantly in their study (6). Comparing the proportion in Stages A and B/C between the students in our population and the 30–39-year group, the population in our study has less high classifications. This is in line with the further development of the proportion between the stages with increasing age in the Morbach et al. study. Barbaresko et al. describe lifestyle behavior associated with CV risks (9). A more careful lifestyle between 20 and 30 years old may change the slope of the curve at higher ages. In this context, it was unexpected, that in this physically active and health-related population, obesity, smoking, and alcohol/drug abuse were dominant risk factors in Stage A. Secondary prevention is needed in this group to create awareness for future health and set medical basics for an individual lifestyle change.

Four participants were set into Stage C. This was a high number compared to the Morbach et al. study, whose population was randomly selected residents of a city in Germany and had no Stage C from 30 to 49 years out of 2,473 participants. Possibly exaggerated sports can have adverse effects on CV health even at a younger age. Because of the small sample size, more research is needed to investigate this phenomenon. However, screening of sports-related groups even in recreational sports is important. This is in line with the analysis of Bohm et al. investigating sports-related sudden cardiac arrest in young adults (8).

The results of the motor performance tests were predominantly not significant between the stages. This was expected, in this age group and their fitness level, shown in the VO2peak values. Additionally, the risk group stages were at a low level, with almost no functional disabilities. Nevertheless, there was a significant difference between the stages in HGS. This result is not more than an indicator for further research, because of the low number of cases in Stage C, but possibly there is an association between the muscular strength of the hand and the risk group classification.

### 4.3 Associations between the biomarkers

Body size, mass, and composition had a significant influence on almost all screening values. The percentage value of fat tissue was negatively correlated with almost all health parameters, particularly CPET, blood pressure and motor performance. The most used calculated value for anthropometric data is the BMI, but as described it is important to take fat and muscle markers into account despite the correlations. As body composition is a result of lifestyle, these screenings are highly important.

Likewise expected was the correlation of the CPET measurements with the results of the motor performance screenings but the plank and the tapping test were independent, because they focus on different capabilities. The different motor performance tests focus on specific capabilities. Despite the correlations, the screenings allowed specific recommendations for the participants.

The concept of the screening test battery was to generate a holistic overall picture of the participants, with a main focus on CV risk factors. The heat map showed no redundant tests. Nevertheless, it is a cost-intensive and time-consuming procedure. For further work, it could be interesting to search for cost- and time-efficient first-level indicators for risk group identification on a large scale.

## 5 Conclusion

Secondary prevention in Germany has a gap for young adults (20–30 years). CV problems predominantly start at the age of 30 and increase with age. A risk group identification according to ACCF/AHA stages in the age of 20–30 years is meaningful. The Stage A and B prevalence for HF in the study population was similar to other studies, which analyzed the next age groups (30–40 years). Living a healthy lifestyle during the early stages of life leads to long-term health benefits and reduces the risk of cardiovascular events in older age. Screenings for freshmen at universities are the perfect time and place. This screenings sensitize for CV risks and lifestyle and detect HF risks at an early age. Moreover, we found a higher prevalence of Stage C in the sports-related study population compared to other studies. The risk for HF could be elevated in sports-related educational institutions. The high prevalence of underweight participants, especially in the female subgroup could also be a specific problem in sports-related groups. Even if not related to CV risks, the awareness of being underweight in combination with offers for a lifestyle change can prevent risks in the future. The same applies to low blood pressure in the female subgroup.

## 6 Limitations

In contrast to other studies using the stage model of ACCF/AHA, we did not use blood samples for the risk group identification. The ethical approval of this study did not include any invasive measurements. An extended test battery compensated for this. However, this test battery is time-consuming (about 2.5 h per participant) and cost-intensive.

## 7 Future research

Secondary prevention is needed in the group of young adults to create awareness for future health and set medical basics for a healthy lifestyle over the lifespan. In the future, the results can help to design and implement needs-orientated and target-group specific prevention programs for the healthy development of adolescents and young adults at universities. For this purpose, the screening needs to be expanded and should include students from other disciplines.

## Data Availability

The raw data supporting the conclusions of this article will be made available by the authors, without undue reservation.
