# Corrigendum: TUM4Health, a holistic student health promotion program. Screening of cardiovascular risk factors in university students

**DOI:** 10.3389/fcvm.2024.1540284

**Published:** 2024-12-12

**Authors:** Klaus Christian Haggenmüller, Barbara Reiner, Renate Maria Oberhoffer, Nils Olson, Jochen Weil, Thorsten Schulz

**Affiliations:** ^1^ Chair of Preventive Pediatrics, Technical University of Munich, Munich, Germany; ^2^ German Heart Centre Munich, Hospital of the Technical University of Munich, Munich, Germany

**Keywords:** cardiovascular risk factors, ACCF/AHA practice guideline, sports & exercise medicine, CPET cardiopulmonary exercise testing, echocardiography, electrocardiography (ECG), biomarkers

A Corrigendum on TUM4Health, a holistic student health promotion program. Screening of cardiovascular risk factors in university students By Haggenmüller KC, Reiner B, Oberhoffer RM, Olson N, Weil J and Schulz T. (2024) Front. Cardiovasc. Med. 11:1428457. doi: 10.3389/fcvm.2024.1428457

In the published article, there was an error in the article title. Instead of “TUM4Health, a holistic student health prevention program. Screening of cardiovascular risk factors in university students”, it should be “TUM4Health, a holistic student health promotion program. Screening of cardiovascular risk factors in university students”.

The authors apologize for this error and state that this does not change the scientific conclusions of the article in any way. The original article has been updated.

