# Supplementary material for: TUM4Health, a holistic student health promotion program. Screening of cardiovascular risk factors in university students
Source: Front Cardiovasc Med. 2024 Nov 27;11:1428457. doi: 10.3389/fcvm.2024.1428457 (PMC11632104; doi:10.3389/fcvm.2024.1428457)
Supplement: Supplementary file 3 [file Table3.pdf]

Gesund durchs Studium	male			female			male	female
Motor Skills	Quartil	Median	Quartil	Quartil	Median	Quartil	N, av. ,std.	N, av. ,std.
Counter Movement Jump [cm]	34	39	43	23	26	29	151, 39±6	266, 26±4
Drop Jump [cm]	21	27	33	17	21	24	152, 27±9	266, 21±6
Handgrip right [kg]	43	48	54	28	32	35	150, 49±8	260, 31±5
Handgrip left [kg]	41	47	52	27	31	34	149, 48±9	259, 31±5
Tapping [n]	58	65	72	52	58	62	154, 65±11	268, 57±9
Standing Long Jump [cm]	214	227	242	159	171	188	127, 222±41	242, 172±28
Plank [s]	149	187	253	121	152	192	126, 204±79	240, 166±83
