# Supplementary material for: TUM4Health, a holistic student health promotion program. Screening of cardiovascular risk factors in university students
Source: Front Cardiovasc Med. 2024 Nov 27;11:1428457. doi: 10.3389/fcvm.2024.1428457 (PMC11632104; doi:10.3389/fcvm.2024.1428457)
Supplement: Supplementary file 4 [file Datasheet1.pdf]

Table of motor performance check-up tests

These tests involve maximum muscular exertion. A standardized warm-up program of 15 minutes of cycling on a bicycle ergometer at 75 watts was used to minimize the risk of injury. It's worth noting that the sports motor tests used in this context are well-established methods in the literature and do not place any additional stress on the test subjects beyond the routine standard diagnostics.

**Counter-Movement-Jump (CMJ)** 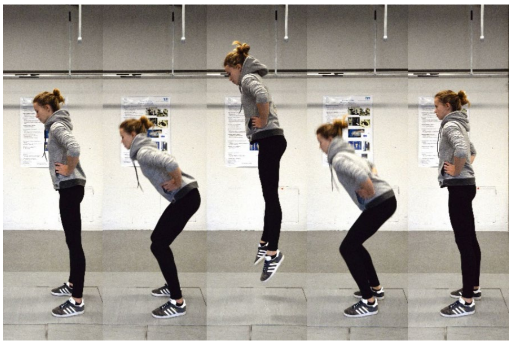	The maximum vertical strength of an individual can be measured by calculating their jumping force using contemplas force plates, manufactured by CONTEMPLAS GmbH in Kempten, Germany. Flight time is used to assess the maximum jump height, and interesting parameters include maximum force and flight time (van Hooren & Zolotarjova, 2017). The participants were instructed to stand upright with their hands on their hips, kneel, jump, and maintain their upright position after landing.
**Drop-Jump (DJ)** 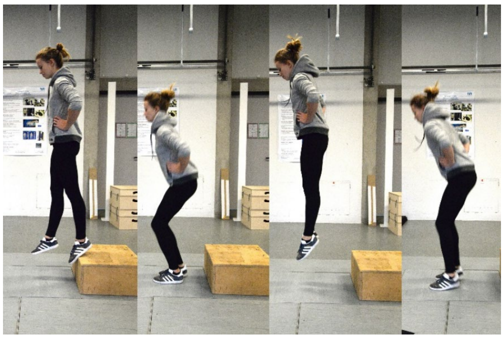	The test used to measure reaction force was the drop jump, which was measured equally via the contemplas force plate. Maximum force, flight time, and contact time were recorded, with only attempts with a contact time under 200 ms being considered valid (Xu et al., 2023). During the test, participants stood with one foot on a 30 cm box, with their hands on their hips and the second foot over the edge of the box. They then dropped down and jumped immediately after making contact, before landing and standing upright.
**Standing-Long-Jump (SLJ)** 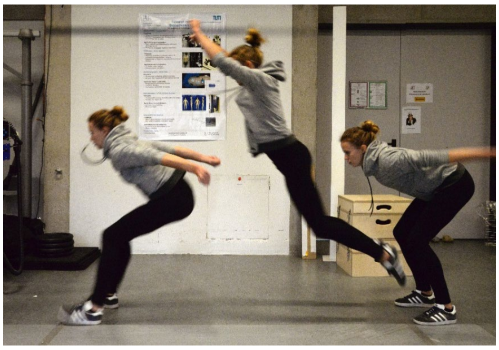	The maximum horizontal strength of the participants was measured using a standing long jump test. The outcome of the test was the distance of the jump, measured in centimeters from the starting line to the heel of the landing foot closest to the starting line, using a tape measure. To perform the test, the participants stood with their toes at the starting line, bent their knees, and jumped forward using an arm swing. They then came to a stop after landing. This test method was described in a study conducted by Castro-Piñero et al. (Castro-Piñero et al., 2010).

**Handgrip Strength (HGS)**<br>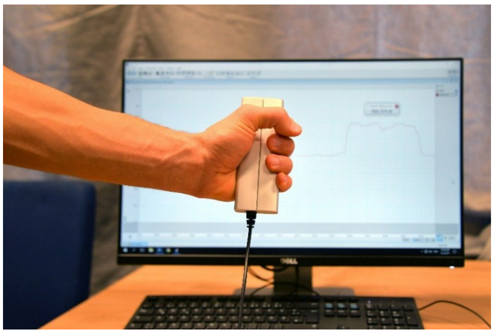	Handgrip strength is a measure used to evaluate muscular strength in sports and health contexts. It is also considered a possible biomarker for overall muscular strength (Leong et al., 2015). The strength is measured using an MLT004/ST Grip Force Transducer manufactured by ADInstruments in New Zealand. The device records strength in Newton over time, and the final result is presented in kilograms. During the test, participants stand with their elbows at a 90-degree angle close to the body and perform two trials on each side.
**Tapping Test**<br>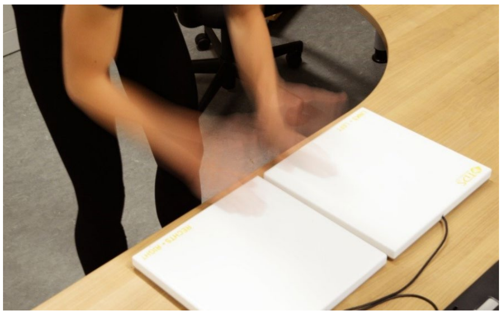	The tapping test is a method used to evaluate neuromuscular ability and sensory-motor speed (Austin et al., 2015). To measure speed force, a 5-second tapping test is performed using a contemplas contact device (CONTEMPLAS GmbH, Kempten, Germany). The test involves hitting two contact plates alternately with the right and left hand, and the outcome is determined by the number of contacts achieved in 5 seconds. The participants stand in front of a desk with the plates during the test.
**Plank Test**<br>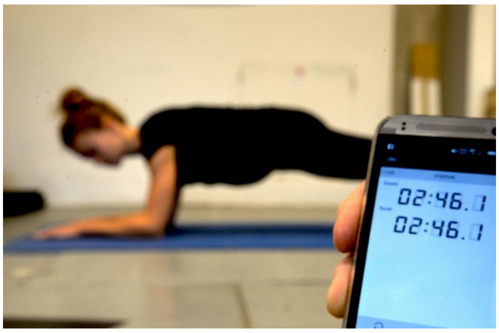	The level of core stability and spinal health can be determined by performing a plank test, as outlined by Laurson et al. (Laurson et al., 2022). This test involves measuring the duration for which a person can maintain the plank position without any movement. To perform the test, participants must get into position with their elbows under their shoulders, forming a straight line from head to heels, and with no hand contact allowed.

References

Austin, D., McNames, J., Klein, K., Jimison, H. & Pavel, M. (2015). A statistical characterization of the finger tapping test: modeling, estimation, and applications. *IEEE Journal of Biomedical and Health Informatics*, *19*(2), 501–507. https://doi.org/10.1109/jbhi.2014.2384911

Castro-Piñero, J., Ortega, F. B., Artero, E. G., Girela-Rejón, M. J., Mora, J., Sjöström, M. & Ruiz, J. R. (2010). Assessing muscular strength in youth: usefulness of standing long jump as a general index of muscular fitness. *Journal of strength and conditioning research*, *24*(7), 1810–1817. https://doi.org/10.1519/JSC.0b013e3181ddb03d

Laurson, K. R., Baptista, F., Mahar, M. T., Welk, G. J. & Janz, K. F. (2022). Designing Health-referenced Standards for the Plank Test of Core Muscular Endurance. *Measurement in Physical Education and Exercise Science*, *26*(4), 344–351. https://doi.org/10.1080/1091367X.2021.2016409

Leong, D. P., Teo, K. K., Rangarajan, S., Lopez-Jaramillo, P., Avezum, A., Orlandini, A., Seron, P., Ahmed, S. H., Rosengren, A., Kelishadi, R., Rahman, O., Swaminathan, S., Iqbal, R., Gupta, R., Lear, S. A., Oguz, A., Yusoff, K., Zatonska, K., Chifamba, J., . . . Yusuf, S. (2015). Prognostic value of grip strength: findings from the Prospective Urban Rural Epidemiology (PURE) study. *Lancet (London, England)*, *386*(9990), 266–273. https://doi.org/10.1016/S0140-6736(14)62000-6

van Hooren, B. & Zolotarjova, J. (2017). The Difference Between Countermovement and Squat Jump Performances: A Review of Underlying Mechanisms With Practical Applications. *Journal of strength and conditioning research*, *31*(7), 2011–2020. https://doi.org/10.1519/JSC.0000000000001913

Xu, J., Turner, A., Comfort, P., Harry, J. R., McMahon, J. J., Chavda, S. & Bishop, C. (2023). A Systematic Review of the Different Calculation Methods for Measuring Jump Height During the Countermovement and Drop Jump Tests. *Sports Medicine*, *53*(5), 1055–1072. https://doi.org/10.1007/s40279-023-01828-x
