# Supplementary material for: TUM4Health, a holistic student health promotion program. Screening of cardiovascular risk factors in university students
Source: Front Cardiovasc Med. 2024 Nov 27;11:1428457. doi: 10.3389/fcvm.2024.1428457 (PMC11632104; doi:10.3389/fcvm.2024.1428457)
Supplement: Supplementary file 2 [file Table2.pdf]

Gesund durchs Studium	male			female			male	female
Medical Screening	Quartil	Median	Quartil	Quartil	Median	Quartil	N, av. ,std.	N, av. ,std.
BMI [kg/m²]	22	23	25	20	21	23	220, 23±2	376, 21±2
Body Fat [%]	9	11	14	18	22	25	214, 12±4	358, 21±6
Skeletal Muscle Mass [kg]	35	38	41	23	25	28	214, 38±6	358, 26±4
IVC% [% norm value]	89	96	105	93	99	107	168, 97±11	312, 100±12
FVC% [% norm value]	91	98	106	89	96	103	189, 99±12	339, 96±11
FEV% [% norm value]	90	96	104	88	95	102	189, 97±11	339, 95±10
VO2peak [ml/min/kg]	45	50	54	37	40	44	203, 50±7	241, 41±6
Pmax [kg]	275	303	337	189	215	238	192, 305±47	325, 214±36
Central Blood Pressure systole [mmHg]	107	117	124	100	106	113	199, 117±12	335, 106±9
Central Blood Pressure diastole [mmHg]	68	73	78	67	72	77	200, 73±9	335, 72±8
Pulse-wave [m/s]	5.0	5.4	5.7	4.7	4.9	5.1	193, 5.3±0.5	334, 4.9±0.3
BMI (Body Mass Index), IVC (Inspiratory Vital Capacity), FVC (Forced Vital Capacity), FEV (Forced Expiratory Volume), Pmax (Power Maximum)								
